# Study on roles of anaplerotic pathways in glutamate overproduction of *Corynebacterium glutamicum *by metabolic flux analysis

**DOI:** 10.1186/1475-2859-6-19

**Published:** 2007-06-23

**Authors:** Tomokazu Shirai, Koki Fujimura, Chikara Furusawa, Keisuke Nagahisa, Suteaki Shioya, Hiroshi Shimizu

**Affiliations:** 1 Department of Biotechnology, Graduate School of Engineering, Osaka University, Japan; 2 Department of Bioinformatic Engineering, Graduate School of Information Science and Technology, Osaka University, Japan

## Abstract

**Background:**

*Corynebacterium glutamicum *has several anaplerotic pathways (anaplerosis), which are essential for the productions of amino acids, such as lysine and glutamate. It is still not clear how flux changes in anaplerotic pathways happen when glutamate production is induced by triggers, such as biotin depletion and the addition of the detergent material, Tween 40. In this study, we quantitatively analyzed which anaplerotic pathway flux most markedly changes the glutamate overproduction induced by Tween 40 addition.

**Results:**

We performed a metabolic flux analysis (MFA) with [1-^13^C]- and [U-^13^C]-labeled glucose in the glutamate production phase of *C. glutamicum*, based on the analysis of the time courses of ^13^C incorporation into proteinogenic amino acids by gas chromatography-mass spectrometry (GC-MS). The flux from phosphoenolpyruvate (PEP) to oxaloacetate (Oxa) catalyzed by phosphoenolpyruvate carboxylase (PEPc) was active in the growth phase not producing glutamate, whereas that from pyruvate to Oxa catalyzed by pyruvate carboxylase (Pc) was inactive. In the glutamate overproduction phase induced by the addition of the detergent material Tween 40, the reaction catalyzed by Pc also became active in addition to the reaction catalyzed by PEPc.

**Conclusion:**

It was clarified by a quantitative ^13^C MFA that the reaction catalyzed by Pc is most markedly increased, whereas other fluxes of PEPc and PEPck remain constant in the glutamate overproduction induced by Tween 40. This result is consistent with the previous results obtained in a comparative study on the glutamate productions of genetically recombinant Pc- and PEPc-overexpressing strains. The importance of a specific reaction in an anaplerotic pathway was elucidated at a metabolic level by MFA.

## Background

A coryneform bacterium, *Corynebacterium glutamicum*, which was isolated in the 1950s [1], is particularly able to produce large amounts of amino acids, such as glutamate and lysine. In glutamate fermentation, it is well known that there are some triggers of glutamate overproduction in *C. glutamicum*: the depletion of biotin, which is required for cell growth [2], the addition of detergent [3], the addition of lactam antibiotics, such as penicillin [4], and the temperature upshift of fermentation [5]. With these triggers a marked change at the final branch point, 2-oxoglutarate in the TCA cycle, was observed owing to a decrease in 2-oxoglutarate dehydrogenase complex (ODHC) activity [6-8]. Recently, it has been reported that the deletion of *odhA *causes glutamate overproduction [9]. Furthermore, it has been clear that the pentose phosphate pathway activity decreases when glutamate production is induced [10].

However, the mechanism of the flux redistribution of 2-oxoglutarate in the TCA cycle is not sufficient for explaining a change in the production activity of glutamate. To overproduce glutamate, oxaloacetate has to be supplied through anaplerotic pathways. Therefore, the changes in fluxes in the anaplerotic pathways are also important for clarifying the mechanism of glutamate overproduction. *C. glutamicum *has a complex set of anaplerotic pathways for supplying oxaloacetate, i.e., reactions from phosphoenolpyruvate (PEP) to oxaloacetate catalyzed by phosphoenolpyruvate carboxylase (PEPc) [11,12], from pyruvate (Pyr) to oxaloacetate catalyzed by pyruvate carboxylase (Pc) [13], from oxaloacetate to PEP catalyzed by PEP carboxykinase (PEPck) [14], and from malate to Pyr catalyzed by malic enzymes [15,16]. Peters-Wendisch *et al*. observed that the ability of *C. glutamicum *to produce glutamate is highly dependent on the Pc activity, using recombinant strains with the overexpression and deficiency of the Pc activity [17]. By comparing the final amount of glutamate production of the recombinant strain of Pc with that of the parental strain, Pc was considered important from a molecular biological view [18]. However, the metabolic fluxes of anaplerotic pathways under glutamate-overproducing conditions have not yet directly been measured.

For the flux analysis of complex pathways, such as anaplerosis, a precise and quantitative metabolic flux analysis involving a ^13^C isotope labeling experiment (^13^C MFA) is performed. For this experiment, the ^13^C-labeled glucose is generally added into a medium and cells are harvested in the log phase. The fractional ^13^C-labeling of proteinogenic amino acids is performed by gas chromatography-mass spectrometry (GC-MS) and nuclear magnetic resonance (NMR). Using this method, metabolic fluxes including anaplerosis can be estimated under the conditions of a continuous culture or mid-log phase, where it is easily possible to establish a steady state and harvest cells [16,18-23]. In the lysine production of *C. glutamicum*, ^13^C MFAs have successfully been performed to analyze the intracellular fluxes in continuous cultures [24,25].

However, there are no reports on the detailed quantitative analyses of anaplerosis based on the ^13^C MFA in the glutamate production phase of *C. glutamicum*, owing to the difficulties of setting the experimental conditions up. After the induction of glutamate production by triggering effects, cell growth is almost stopped in wild-type strains [8]. Thus, even when cells are cultured in a medium with a ^13^C-labeled substrate, the fractional ^13^C-labelings of proteinogenic amino acids from cells in the production phase do not always represent the metabolic flux distribution in the production phase. In this case, it mainly represents the metabolic flux distribution in the log growth phase before the triggering effects, since intracellular proteins are predominantly synthesized in the growth phase. Therefore, to extract pure information about metabolic fluxes in the glutamate production phase, a ^13^C-labeled substrate must be added into the medium after cells enter the glutamate production phase.

In this study, the direct quantitative analysis of metabolic fluxes by ^13^C MFA was performed in the glutamate production phase of *C. glutamicum*. The important points for the ^13^C MFA in the glutamate production phase are as follows: (1) ^13^C of metabolites should be converted from the ^13^C-labeled substrate (glucose) taken up in the glutamate production phase only and (2) reaching the plateau of the incorporation rate of ^13^C into proteinogenic amino acids in this phase should be confirmed. We established an experimental and analytical method for the ^13^C MFA in the glutamate production phase induced by Tween 40 addition as follows.

*C. glutamicum *was cultivated on natural glucose in the growth phase, and then glutamate production was induced by Tween 40 addition in the mid-log growth phase. The amount of Tween 40 added was precisely adjusted to achieve both stable growth and glutamate production, as ^13^C was incorporated into proteinogenic amino acids in the production phase. Subsequently, ^13^C-labeled glucose was added in the glutamate production phase, and the time course of the ^13^C labeling pattern of each amino acid was measured by GC-MS. It was confirmed that the ^13^C labeling patterns of the amino acids reach a plateau and the steady state by comparing them with that at each sampling time. A computational algorithm for precise ^13^C MFAs [26] was applied to the determination of metabolic fluxes by fitting ^13^C labeling patterns.

In this study, two different glutamate production activities were induced by the addition of two different amounts of Tween 40. The ^13^C MFA results between the growth and two different glutamate production were comparatively analyzed. From the obtained results, the roles of anaplerotic pathways (Pc, PEPc, and PEPck) in the glutamate overproduction induced by Tween 40 addition in *C. glutamicum *were clarified.

## Results

### Glutamate productions of *C. glutamicum *under two Tween 40 addition conditions

**Figure 1:**
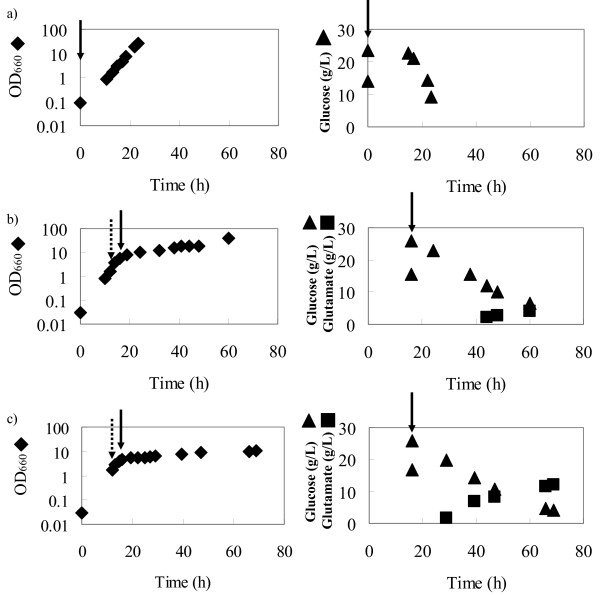
**Time courses of cell growth (OD_660_), glucose consumption, and glutamate production of *C. glutamicum***. Figs. 1-a, -b, and -c indicate the fermentations with Tween 40 additions using the final concentrations of 0 (without addition), 0.5, and 0.8 mg/mL, respectively. Diamonds, triangles, and squares indicate OD_660_, glucose, and glutamate, respectively. Dark and dotted arrows indicate the points of ^13^C glucose ([1-^13^C] and [U-^13^C] glucose) and Tween 40 additions, respectively.

The fermentation of *C. glutamicum *was performed under two Tween 40 addition conditions: final concentrations of 0.5 and 0.8 mg/mL. The obtained results are shown in Fig. 1. It was obvious that the glutamate production in the fermentation under the 0.8 mg/mL of Tween 40 addition condition (Fig. 1-c) is larger than in the fermentation under 0.5 mg/mL of the Tween 40 addition condition (Fig. 1-b). In the fermentation without Tween 40 addition, no glutamate was produced into the medium (Fig. 1-a). Specific rates in the growth and the glutamate production phases are shown in Table 1 (all units: per hour). The glutamate production fluxes in the fermentations under the two Tween 40 addition conditions were 20 (Fig. 1-b) and 68 (Fig. 1-c) with respect to the glucose uptake of 100. The amount of glutamate overproduced was proportional to that of Tween 40 added.

**Table 1:** Specific rates in growth and two glutamate production phases of *C. glutamicum*.

	Amount of Tween 40 addition (final concentration (mg/ml))		
	
	0	0.5	0.8
growth rate (μ)	0.25	0.04	0.01
consumption rate (ν)	0.55	0.14	0.17
production rate (ρ)	-	0.03	0.12

### Observation of ^13^C incorporation into each proteinogenic amino acid by GC-MS

**Figure 2:**
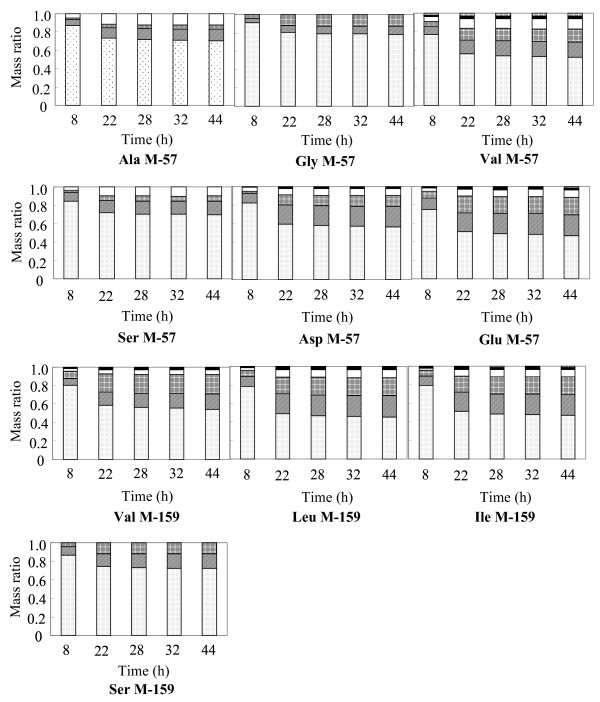
**Time courses of mass isotopomer distributions of proteinogenic amino acids in fermentation after addition of ^13^C-labeled glucose, where glutamate production was 20 with respect to glucose uptake of 100**. The data (*m*0, *m*1 ..., *m*n indicating the mass isotopomer distributions obtained from mass spectral intensity ratios, as previously described [22]) at each sampling time were summed up to unity from the bottom. Time indicates that after ^13^C glucose addition. The M-57 and M-159 of each amino acid indicate mass ion fragment groups with high intensities, and thus, these data were used for ^13^C MFA.

For the ^13^C MFA of *C. glutamicum *in the glutamate production phase, it was necessary to obtain the information of ^13^C of each amino acid converted in the glutamate production phase. In this study, the time course of ^13^C incorporation into each amino acid was measured by GC-MS analysis. The obtained result is shown in Fig. 2, where the glutamate production flux was 20 with respect to the glucose uptake of 100 (Fig. 1-b). The GC-MS data of all the amino acids were not changed after 44 h of ^13^C-labeled glucose addition (60 h in the fermentation) and reached a plateau. Therefore, these GC-MS data were used for the ^13^C MFA in the glutamate production phase. Also, for the ^13^C MFA in the production phase in the fermentation with a glutamate production flux of 68 (Fig. 1-c), the GC-MS data were used after 53 h since ^13^C-labeled glucose had been added (69 h in the fermentation).

### ^13^C MFAs in growth phase and two glutamate production phases

**Figure 3:**
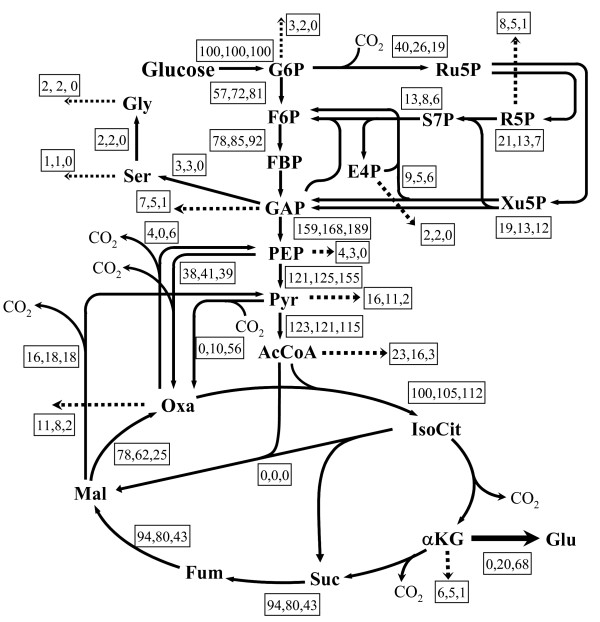
**Metabolic fluxes in *C. glutamicum *in growth and production phases of two different glutamate production activities**. Dotted arrows indicate fluxes for biomass. Left, middle, and right values in boxes indicate fluxes in the growth and production phases, where glutamate fluxes were 20 and 68, respectively. In this study, the fluxes with backward (exchange) reactions, i.e., those in glycolysis, the pentose phosphate pathway, the latter steps of the TCA cycle (succinate → oxaloacetate), and C1 metabolisms, are shown as net values [22]. Abbreviations: Gly, glycine; Ser, serine; Glu, glutamate; G6P, glucose-6-phosphate; F6P, fructose-6-phosphate; FBP, fructose-1,6-bisphosphate; GAP, glyceraldehyde-3-phosphate; PEP, phosphoenolpyruvate; Pyr, pyruvate; Ru5P, ribulose-5-phosphate; R5P, ribose-5-phosphate; Xu5P, xylulose-5-phosphate; S7P, sedoheptulose-7-phosphate; E4P, erythrose-4-phosphate; AcCoA, acetyl-CoA; IsoCit, isocitrate; αKG, 2-oxoglutarate; Suc, succinate; Fum, fumarate; Mal, malate; Oxa, oxaloacetate.

The ^13^C MFA in the growth phase was performed by a previously reported method [26], where ^13^C-labeled glucose was added at the beginning of fermentation, that is, the GC-MS data in the log growth phase was used for analysis (22 h). The ^13^C MFAs in the two glutamate production phases were performed using the GC-MS data mentioned in the previous section. The GC-MS data of *C. glutamicum *in the growth phase and two glutamate production phases are shown in Tables 2, 3, 4, respectively. The measurement of each sample was performed five times, and all data errors (standard deviation divided by mean values) were below 2%. The ^13^C MFA results in the growth phase and two glutamate production phases are shown in Fig. 3. For the result in the growth phase, cross validation was performed using the NMR data shown in Table 5. For the ^13^C MFA results in the two production phases, the results based on the ^13^C labeling patterns of proteinogenic amino acids were verified using the GC-MS data of intermediate metabolites (pyruvate, succinate, and fumarate) because of difficulty in cross validation using the NMR data due to a small number of cells in the production phase, as shown in Tables 6 and 7.

**Table 2:** Mass isotopomer distributions of amino acids in growth phase of *C. glutamicum*.

		*m*0	*m*1	*m*2	*m*3	*m*4	*m*5
Ala-57	measured	0.677	0.128	0.040	0.155		
	estimated	0.665	0.138	0.041	0.156		
Gly-57	measured	0.753	0.076	0.171			
	estimated	0.754	0.076	0.170			
Val-57	measured	0.472	0.162	0.158	0.138	0.035	0.034
	estimated	0.465	0.178	0.164	0.139	0.026	0.028
Ser-57	measured	0.654	0.154	0.047	0.146		
	estimated	0.669	0.136	0.047	0.147		
Phe-57	measured	0.310	0.165	0.135	0.151	0.103	0.061
	estimated	0.308	0.174	0.141	0.153	0.106	0.057
Asp-57	measured	0.495	0.237	0.128	0.106	0.033	
	estimated	0.506	0.227	0.155	0.099	0.013	
Glu-57	measured	0.389	0.226	0.221	0.111	0.039	0.013
	estimated	0.386	0.243	0.230	0.099	0.032	0.009
His-57	measured	0.411	0.209	0.130	0.103	0.058	0.058
	estimated	0.422	0.243	0.123	0.097	0.053	0.054
Val-159	measured	0.496	0.156	0.258	0.054	0.036	
	estimated	0.489	0.170	0.265	0.044	0.032	
Leu-159	measured	0.361	0.243	0.239	0.105	0.040	0.012
	estimated	0.357	0.256	0.240	0.103	0.035	0.009
Ile-159	measured	0.398	0.224	0.229	0.099	0.036	0.013
	estimated	0.386	0.243	0.230	0.099	0.032	0.009
Ser-159	measured	0.691	0.131	0.178			
	estimated	0.702	0.131	0.166			
Phe-159	measured	0.350	0.157	0.194	0.109	0.093	0.046
	estimated	0.323	0.170	0.212	0.103	0.103	0.042

The underlined values are those used for ^13^C MFA based on the threshold (value > 0.06, error < 3%). -57 and -159 in amino acids indicate [M-57] and [M-159] groups of GC-MS analysis, whose ion fragments have high intensities [26, 42, 43].

**Table 3:** Mass isotopomer distributions of amino acids in glutamate production phase of *C. glutamicum*, where glutamate flux was 20 with respect to glucose uptake of 100.

		*m*0	*m*1	*m*2	*m*3	*m*4	*m*5
Ala-57	measured	0.702	0.129	0.047	0.122		
	estimated	0.706	0.135	0.041	0.117		
Gly-57	measured	0.782	0.087	0.131			
	estimated	0.790	0.083	0.127			
Val-57	measured	0.529	0.166	0.139	0.111	0.029	0.026
	estimated	0.527	0.178	0.148	0.111	0.019	0.017
Ser-57	measured	0.701	0.139	0.058	0.102		
	estimated	0.708	0.139	0.058	0.095		
Phe-57	measured	0.404	0.167	0.123	0.121	0.082	0.048
	estimated	0.381	0.181	0.135	0.135	0.088	0.041
Asp-57	measured	0.572	0.215	0.117	0.077	0.019	
	estimated	0.575	0.211	0.130	0.075	0.008	
Glu-57	measured	0.466	0.226	0.196	0.077	0.028	0.007
	estimated	0.462	0.236	0.202	0.073	0.021	0.005
His-57	measured	0.487	0.199	0.117	0.100	0.048	0.036
	estimated	0.492	0.228	0.112	0.082	0.035	0.045
Val-159	measured	0.550	0.164	0.210	0.050	0.026	
	estimated	0.557	0.163	0.227	0.032	0.021	
Leu-159	measured	0.452	0.231	0.198	0.083	0.028	0.008
	estimated	0.430	0.253	0.213	0.076	0.023	0.005
Ile-159	measured	0.471	0.225	0.194	0.076	0.026	0.008
	estimated	0.462	0.236	0.202	0.073	0.021	0.005
Ser-159	measured	0.721	0.158	0.121			
	estimated	0.747	0.138	0.115			
Phe-159	measured	0.418	0.169	0.167	0.092	0.077	0.037
	estimated	0.402	0.170	0.200	0.085	0.085	0.029

The underlined values are those used for ^13^C MFA based on the threshold (value > 0.06, error < 3%).-57 and -159 in amino acids indicate [M-57] and [M-159] groups of GC-MS analysis, whose ion fragments have high intensities [26, 42, 43].

**Table 4:** Mass isotopomer distributions of amino acids in glutamate production phase of *C. glutamicum*, where glutamate flux was 68 with respect to glucose uptake of 100.

		*m*0	*M*1	*m*2	*m*3	*m*4	*m*5
Ala-57	measured	0.676	0.145	0.025	0.154		
	estimated	0.670	0.140	0.042	0.148		
Gly-57	measured	0.768	0.073	0.158			
	estimated	0.769	0.072	0.159			
Val-57	measured	0.463	0.189	0.164	0.133	0.021	0.030
	estimated	0.472	0.179	0.164	0.134	0.025	0.026
Ser-57	measured	0.651	0.170	0.082	0.097		
	estimated	0.657	0.163	0.087	0.093		
Phe-57	measured	0.324	0.160	0.150	0.150	0.104	0.051
	estimated	0.334	0.169	0.139	0.141	0.104	0.055
Asp-57	measured	0.586	0.185	0.102	0.116	0.010	
	estimated	0.582	0.183	0.104	0.117	0.014	
Glu-57	measured	0.435	0.211	0.227	0.091	0.028	0.009
	estimated	0.436	0.207	0.229	0.088	0.031	0.010
His-57	measured	0.477	0.223	0.099	0.073	0.032	0.080
	estimated	0.466	0.232	0.099	0.072	0.047	0.071
Val-159	measured	0.484	0.162	0.272	0.050	0.031	
	estimated	0.495	0.168	0.263	0.042	0.031	
Leu-159	measured	0.346	0.256	0.246	0.110	0.032	0.010
	estimated	0.359	0.258	0.237	0.103	0.034	0.009
Ile-159	measured	0.446	0.201	0.228	0.086	0.033	0.007
	estimated	0.436	0.207	0.229	0.088	0.031	0.010
Ser-159	measured	0.693	0.206	0.101			
	estimated	0.689	0.203	0.108			
Phe-159	measured	0.351	0.164	0.212	0.084	0.105	0.040
	estimated	0.350	0.162	0.214	0.084	0.105	0.037

The underlined values are those used for ^13^C MFA based on the threshold (value > 0.06, error < 3%).-57 and -159 in amino acids indicate [M-57] and [M-159] groups of GC-MS analysis, whose ion fragments have high intensities [26, 42, 43].

**Table 5:** Measured values of amino acids by NMR analysis in growth phase of *C. glutamicum*.

Amino Acid	Pre.		*I*_s_	*I*_d1_	*I*_d2_	*I*_dd_	*I*_t_
Ser C3	GAP C3	measured	0.37	0.63			
		estimated	0.35	0.65			
Gly C2	GAP C2	measured	0.17	0.83			
		estimated	0.18	0.82			
Ala C2	Pyr C2	measured	0.09	0.06	0.13	0.72	
		estimated	0.11	0.06	0.11	0.72	
Ala C3	Pyr C3	measured	0.33	0.67			
		estimated	0.34	0.66			
Val C4	Pyr C3	measured	0.33	0.67			
		estimated	0.34	0.66			
Leu C5	Pyr C3	measured	0.33	0.67			
		estimated	0.34	0.66			
Asp C3	Oxa C3	measured	0.31	0.20	0.28	0.21	
		estimated	0.31	0.21	0.29	0.19	
Thr C3	Oxa C3	measured	0.36	0.54			0.11
		estimated	0.40	0.51			0.09
Thr C4	Oxa C4	measured	0.42	0.58			
		estimated	0.38	0.62			
Glu C3	αKG C3	measured	0.43	0.47			0.10
		estimated	0.44	0.45			0.11
Arg C5	αKG C5	measured	0.18	0.82			
		estimated	0.17	0.83			

Symbols: *I*_s_, *I*_d1_, *I*_d2_, *I*_dd_, and *I*_t _indicate coupling pattern of NMR, that is, singlet, doublet1, doublet2, double-doublet, and triplet, respectively. Abbreviations: Pre., precursor of amino acid. For others, see Fig. 1.

**Table 6:** Mass isotopomer distributions of intermediate metabolites in glutamate production phase 1, where glutamate flux was 20 with respect to glucose uptake of 100.

		*m*0	*m*1	*m*2	*m*3	*m*4
Pyruvate	measured	0.701	0.131	0.055	0.113	
	estimated	0.707	0.135	0.041	0.117	
Succinate	measured	0.581	0.214	0.122	0.074	0.008
	estimated	0.570	0.214	0.134	0.073	0.009
Fumarate	measured	0.561	0.220	0.144	0.065	0.009
	estimated	0.572	0.213	0.133	0.074	0.009

**Table 7:** Mass isotopomer distributions of intermediate metabolites in glutamate production phase 2, where glutamate flux was 68 with respect to glucose uptake of 100.

		*m*0	*m*1	*m*2	*m*3	*m*4
Pyruvate	measured	0.669	0.129	0.052	0.149	
	estimated	0.670	0.140	0.042	0.148	
Succinate	measured	0.551	0.194	0.103	0.124	0.029
	estimated	0.562	0.189	0.127	0.106	0.016
Fumarate	measured	0.576	0.180	0.132	0.089	0.022
	estimated	0.568	0.187	0.120	0.109	0.015

As the glutamate production increased with the amount of Tween 40 added, fluxes in the central metabolic pathway changed. Particularly, the flux distributions between glycolysis and the pentose phosphate pathway around the glucose-6-phosphate (G6P) branch point obviously changed. As the glutamate production increased, the flux distribution in the pentose phosphate pathway decreased (from 40 to 26 and 19).

### Correlation between glutamate production and each flux in anaplerosis

**Figure 4:**
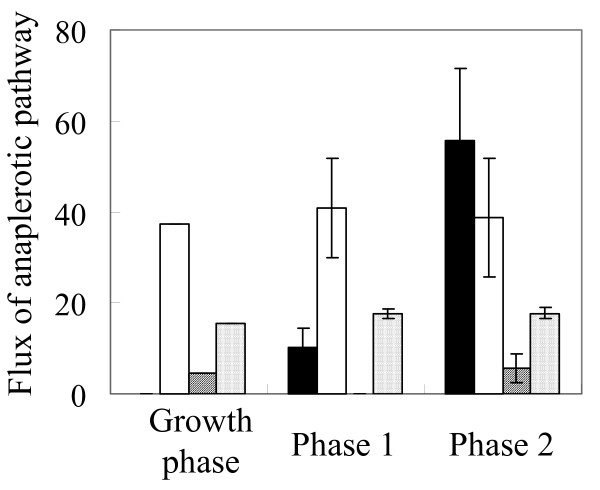
**Comparison of fluxes in anaplerotic pathways in different phases**. Black, white, zebra, and dotted bars indicate the reactions catalyzed by Pc, PEPc, PEPck, and malic enzymes, respectively. Phases 1 and 2 indicate the fermentations with the glutamate production fluxes of 20 and 68, respectively. Flux values of the anaplerosis in three phases (growth, Phase 1, and Phase 2) were as follows: 0, 10, 56 by Pc, 37, 41, 39 by PEPc, 4, 0, 6 by PEPck, and 13, 18, 18 by malic enzymes, respectively. Error bars indicate those with fluxes induced by the errors of GC-MS data, which were obtained from 20 sets of artificial GC-MS data with a variance due to the observed experimental errors (below 2% in this study), as previously described [25].

We determined which reaction in the anaplerotic pathways of *C. glutamicum *has the greatest correlation with the amount of glutamate produced by the quantitative analysis of the ^13^C MFA results. As shown in Fig. 4, reactions from PEP to oxaloacetate catalyzed by PEPc and from malate to pyruvate catalyzed by malic enzymes were mainly active in the growth phase. The flux catalyzed by PEPc, which showed a certain extent value (around 40) in the growth phase, was not significantly changed, even though the condition of fermentation was changed from cell growth to glutamate production by Tween 40 addition. The flux of the reaction from pyruvate to oxaloacetate catalyzed by Pc was almost zero in the growth phase. As glutamate production increased with the amount of Tween 40 added, the flux of this reaction catalyzed by Pc increased. The fluxes of the other anaplerotic pathways from oxaloacetate to PEP catalyzed by PEPck and glyoxylate shunt were almost zero in all cases.

## Discussion

In this study, different glutamate production activities dependent on the amount of Tween 40 added were obtained (Fig. 1). The GC-MS data of amino acids did not change 32–44 h after ^13^C-labeled glucose addition (48–60 h in the fermentation) (Fig. 2). This finding indicates that the condition of ^13^C in each amino acid in the cell reaches a plateau. The ^13^C labeling patterns of the proteinogenic amino acids, that is, the GC-MS data at the time point, indicate the information obtained only in the glutamate production phase. This is also confirmed by verifying the ^13^C MFA results in the two glutamate production phases (Tables 6 and 7). The GC-MS data of proteinogenic amino acids are good agreement with the estimated ones (Tables 3 and 4). Furthermore, the estimated ones are good agreement with the data of precursor ones which are considered to represent the conditions where sample was harvested and the extraction was performed (Tables 6 and 7). This strongly supports that the estimated fluxes using the GC-MS data of proteinogenic amino acids (Tables 3 and 4) represent the condition of the two glutamate production phase. Therefore, ^13^C MFAs in the glutamate production phases by obtaining the time courses of proteinogenic amino acids are considered to be successful.

As the glutamate production of *C. glutamicum *increased, the flux distribution in the pentose phosphate pathway around the glucose-6-phosphate branch point decreased from 40 to 26 and 19, as shown in Fig. 3. These results are similar to the previous study [10], and in contrast to those in the lysine production of *C. glutamicum *[24,25,27-30]. In lysine production, the energy metabolite NADPH, which is mainly produced via the pentose phosphate pathway, is necessary for not only cell growth but also lysine synthesis. Therefore, the flux distribution in the pentose phosphate pathway increases in the lysine production phase, compared with that in the growth phase. Also, in the glutamate production phase, NADPH is necessary for synthesizing glutamate from 2-oxoglutarate (α-KG) catalyzed by glutamate dehydrogenase (GDH). However, NADPH for glutamate synthesis was considered to be sufficiently supplied via a reaction from isocitrate to 2-oxoglutarate in the TCA cycle catalyzed by isocitrate dehydrogenase (ICDH) [31,32]. In lysine production, NADPH has to be supplied via the pentose phosphate pathway, since this reaction in the TCA cycle is related to lysine production. Therefore, as the cell growth decreased with the increase in the amount of Tween 40 added, the amount of NADPH necessary for the syntheses of the cell components decreased; that is, the flux distribution in the pentose phosphate pathway decreased. If glutamate production is more increased, and consequently cell growth was stopped completely as previous described [7,8], the flux on the pentose phosphate pathway would decrease more.

For glutamate production, anaplerotic pathways are essential, as mentioned in Background. In this study, the direct quantitative analysis of the flux changes in the anaplerotic pathways was performed by a ^13^C MFA method in the wild-type strain of *C. glutamicum*, which is different from the previously described method comparing the glutamate productions of the parental and recombinant strains [17]. The malic enzyme activities in three batch fermentations allow us to precisely and independently determine the fluxes of Pc and PEPc in anaplerosis, as previously described [16,21]. This was strongly supported by a 10% increase in the sum of absolute residual errors between the estimated and measured GC-MS data, when metabolic fluxes were re-determined by replacing the flux of Pc with that of PEPc. In the growth phase of *C. glutamicum*, the main active reaction was from PEP to oxaloacetate catalyzed by PEPc (Figs. 3 and 4). Although it might be claimed that this result is in contrast to the previous studies that strains deleted PEPc activity shows no growth defects on glucose [33,34]. In these reports, it is considered that Pc instead of PEPc is active due to the PEPc deletion It was also separately reported that not PEPc but Pc is mainly active in continuous cultures [16,35]. In this study, batch fermentations were performed, and thus, environmental condition around cell such as glucose and ammonia concentrations was quite different from those of continuous culture. In the growth phase in the batch cultures, PEPc is considered to be mainly active. However, in the glutamate production phase, this flux did not increase, even though a higher activity in anaplerosis was required than that in the growth phase. From the results of the quantitative ^13^C MFA in the glutamate production phase, it is considered that this reaction catalyzed by PEPc is not affected by environmental changes due to Tween 40 addition (Fig. 4). Peters-Wendisch and coworkers also found that PEPc is dispensable for glutamate production using a *C. glutamicum *WT (pMF1014) strain with a specific PEPc activity more than 10-fold higher than that of the wild-type strain [17,38]. It is found that the flux of Pc proportionally increases to glutamate production; in contrast, the flux of PEPc remains constant throughout fermentation (Fig. 4). This result directly indicates that the flux of Pc is the most important of those in the anaplerotic pathways in the glutamate overproduction induced by Tween 40 addition. It is considered that the flux of Pc would be more active as glutamate production increases, and simultaneously, cell growth is rarely induced using a larger amount of Tween 40 added than that in this study.

Furthermore, a backward flux from oxaloacetate catalyzed by PEPck, which was considered to be a negative factor for glutamate production, was inactive in both the growth and glutamate production phases of the batch fermentations. In the batch fermentations in this study, malic enzymes is active but PEPck is not active, owing to high ammonium ion concentration compared to that in continuous cultures [36], although this result is in contrast to that in continuous cultures as previously described [16,37]. Thus, the inhibition or attenuation of the PEPck activity of *C. glutamicum *by genetic modification might not be a necessary strategy for improving in glutamate production by Tween 40 addition. This would also be considered to be related to the cases involving malic enzymes and a glyoxylate shunt, because these fluxes did not change and were inactive in all the batch fermentations, as previously described [21,28].

## Conclusion

We established experimental conditions under which both cell growth and glutamate production occurred simultaneously by Tween 40 addition. We quantitatively investigated how fluxes in the central metabolism of *C. glutamicum *change as glutamate production increases. The flux distribution in the pentose phosphate pathway around the glucose-6-phosphate branch point decreased as glutamate production increased. The flux of Pc proportionally increased to glutamate production despite the flux of PEPc remaining constant throughout batch fermentation. This result directly indicates that the flux of Pc is the most important of those in the anaplerotic pathways in the glutamate overproduction induced by Tween 40 addition, and is well consistent with that of the genetic evidence. The quantitative ^13^C MFA in the glutamate production phase in this study indicated which reaction in the anaplerotic pathways should be controlled; that is, what type of cell modification should be performed.

## Methods

### Bacterial strains and medium

*C. glutamicum *AJ-1511 (ATCC13869) was used. The same medium as that employed in our previous study was used for the preculture of the microorganism [26] (per liter deionized water): 40 g of glucose, 30 g of (NH_4_)_2_SO_4_, 3.0 g of Na_2_HPO_4_, 6.0 g of KH_2_PO_4_, 2.0 g of NaCl, 84 mg of CaCl_2_, 3.9 mg of FeCl_3_, 0.9 mg of ZnSO_4_·7H_2_O, 0.3 mg of CuCl_2_·H_2_O, 5.56 mg of MnSO_4_·5H_2_O, 0.1 mg of (NH_4_)_6_MO_7_O_24_·' m4H_2_O, 0.3 mg of Na_2_B_4_O_7_·10H_2_O, 0.4 g of MgSO_4_·7H_2_O, 40 mg of FeSO_4_·7H_2_O, 500 μg of vitamin B_1_·HCl, 0.1 g of EDTA, and 10 μg of biotin. The main culture medium composition was the same as the preculture medium composition except for the initial glucose concentration of 20 g/L. For the ^13^C MFA in the growth phase, the initial glucose (natural abundance of glucose) concentration was 15 g/L (see below and Fig. 1-a).

### Culture conditions

The cultivation of *C. glutamicum *for ^13^C metabolic flux analysis (^13^C MFA) was carried out using a 250-mL jar fermenter with a liquid working volume of 80 mL (ABLE Co., Japan). The culture temperature was 31.5°C. The pH was maintained at 7.2 by adding 2.0 g of CaCO_3 _at the beginning of fermentation. The dissolved oxygen concentration was maintained above 3.0 mg/L by controlling the agitation speed in the range of 400–800 revolutions per minute (rpm). The aeration rate was maintained at 2 L of air volume per liter of liquid volume per minute (vvm). Two types of glutamate production were induced using two different amounts of Tween 40 added, that is, the final concentrations of 0.5 and 0.8 mg/mL. Subsequently, 1 mL of 400 g/L [1-^13^C]-labeled and 1 mL of [U-^13^C]-labeled glucose were added to the batch culture. For the ^13^C MFA in the growth phase, such components were added at the beginning of fermentation (see Fig. 1-a).

### Off-line measurement

Cell concentration was measured at OD_660_. Glucose concentration was measured enzymatically using a glucose analyzer (Model-2700, YSI, USA). Glutamate concentration was measured by a colorimetric method using a glutamate kit (Roche Co., Germany), as previously described [8].

### Sample preparation for GC-MS analysis

For ^13^C labeling experiment, cells were harvested in the log growth and glutamate production phases by centrifugation and then hydrolyzed in 6 M HCl at 105°C for 18 hours. Amino acids were purified and evaporated, as previously described [26,27]. For GC-MS, the amino acids were derivatized prior to their analysis. The dried hydrolysate was derivatized by *N*-(*tert*-butyldimethylsilyl (TBDMS))-*N*-methyl-trifluoroacetamide (MTBSTFA) [39,40], and 1 μL of the sample was injected to the GC-MS system.

For the GC-MS measurements of the ^13^C labeling patterns of the intermediate metabolites considered in this study (pyruvate, succinate, and fumarate), these metabolites were extracted from cells. In this study, the modified method previously described was used [41,42]. Cell broth (5 mL) was harvested at 60 and 69 h in two batch fermentations and immediately dropped in liquid nitrogen for cell quenching. Subsequently, cells were washed twice using 1.0% KCl at 4°C by centrifugation and added to 500 μL of methanol cooled at -20°C for 30 min. After centrifugation, the supernatant was used for GC-MS analysis after evaporation in dried nitrogen stream. The dried residue was redissolved and derivatized for 90 min at 30°C in 100 μL of 20 mg/mL methoxyamine hydrochloride in pyridine. Subsequently, trimethylsilylation (TMS derivatization) was performed for 30 min at 37°C and then for 2 hours at room temperature with 100 μL of *N*-methyl-*N*-(trimethylsilyl)trifluoroacetamide (MSTFA) [42,43]. The sample (1 μL) was injected to the GC-MS system.

### Metabolic flux analysis (MFA)

The metabolic pathways for analyzing the flux distribution in glutamate production and the metabolic reaction model developed here were previously reported [26]. In the main part of the model, the central metabolic pathway including glycolysis, the tricarboxylic acid (TCA) cycle and the pentose phosphate pathway were considered for ^13^C MFA. Furthermore, the anaplerotic pathways (anaplerosis) essential for glutamate production and biosynthesis pathways for a cell were also considered in the model [26]. The fluxes for biomass synthesis of *C. glutamicum *were cited from Marx et al. [27], except for the biosynthesis fluxes of amino acids for proteins, which were calculated based on the content of each amino acid determined by GC-MS as previously described [26]. To evaluate precision of estimated fluxes, standard deviations of fluxes with respect to experimental errors was studied by investigating how much the estimated flux varied with such dispersion of artificial GC-MS data [26]. NMR analysis of the same amino acids was performed to check for consistency with the ^13^C labeling patterns estimated based on the MFA using only GC-MS.

### GC-MS

GC-MS was carried out using a Shimadzu GC-17A gas chromatograph equipped with an InertCap1MS capillary column (25 m × 0.25 mm × 0.25 μm; GL Science, Tokyo) that was directly connected to a JMS-AMSUN200HS mass spectrometer (JEOL, Co., Japan), as previously described [26]. The settings of the measurements of TBDMS amino acid derivatives were used, as previously described [26].

For the measurements of TMS derivatives, an initial oven temperature of 70°C was maintained for 5 min, then increased to 300°C at 10°C/min and maintained for 8 min. The total running time was 35 min. The other settings were the same as those of the measurements of TBDMS derivatives (amino acids). In this study, the ion fragment of [M-15] was measured by GC-MS [43]. The GC-MS data of TMS derivatives were obtained by calculating the ratio of each intensity to the sum of all intensities in the [M-15] groups. For ^13^C MFA, correct mass data, where abundances of natural isotopes were removed from the raw GC-MS data, were used as previously described [44].

## Authors' contributions

TS participated in the design of this study, performing fermentation experiments, the measurements of metabolites by GC-MS and the calculation of fluxes using computers. He analyzed the data and wrote the manuscript.

KF participated in the design of the study and measuring metabolites by GC-MS.

CF participated in the design of the study and reviewed the manuscript.

KN participated in the design of the study and reviewed the manuscript.

SS participated in the design of the study and reviewed the manuscript.

HS participated in the design of the study and reviewed the manuscript.

All authors have read and approved the manuscript.
